# Onset of activity of fluralaner (BRAVECTO™) against *Ctenocephalides felis* on dogs

**DOI:** 10.1186/s13071-014-0567-6

**Published:** 2014-12-04

**Authors:** Janina Taenzler, Christina Wengenmayer, Heike Williams, Josephus Fourie, Eva Zschiesche, Rainer KA Roepke, Anja R Heckeroth

**Affiliations:** MSD Animal Health Innovation GmbH, Zur Propstei, 55270 Schwabenheim, Germany; ClinVet International, Uitsigweg, Bainsvlei, 9338 Bloemfontein, Free State South Africa

**Keywords:** Bravecto™, Chewable tablets, Fluralaner, Onset of activity, Start to kill, Speed of kill, Dog, Flea, *Ctenocephalides felis*, Efficacy

## Abstract

**Background:**

Fluralaner (Bravecto™) is a novel systemic insecticide and acaricide that provides long persistent antiparasitic activity following a single administration at the minimum dose of 25 mg/kg body weight.

**Methods:**

Three negative controlled, randomized studies were conducted in dogs to evaluate the start to kill (1 study) and the speed of flea kill (2 studies) of fluralaner. All dogs were infested prior to treatment with unfed adult *C. felis* fleas. Dogs in the treated groups were administered once orally with fluralaner at a minimum dose of 25 mg/kg body weight, while dogs in the control groups were not treated. Separate control and treatment groups were paired at each time point of flea assessment. Flea counts were performed by combing dogs at either 0.5, 1, 2, or 4 hours after fluralaner treatment to measure the start to kill. To evaluate the speed of flea kill over 12 weeks, flea counts were performed by combing dogs at either 4, 8, 12, or 24 hours after fluralaner treatment and then at 4, 8, 12, or 24 hours after each flea re-infestations performed at 4, 8, and 12 weeks following treatment.

**Results:**

In the start to kill study, the fluralaner activity against fleas started already at 1 hour post-treatment (8% numerical efficacy). At 2 and 4 hours post-treatment, the flea reduction was significant with 36.7% and 88% efficacy, respectively.

In the speed of kill studies, the efficacy against fleas after fluralaner treatment was 80.5% at 4 hours and remained ≥ 99.4% at 8, 12 and 24 hours. After flea re-infestations in weeks 4, 8 and 12, the efficacy at 4 hours was 96.8, 91.4, and 33.5%, respectively. Efficacy at 8, 12 and 24 hours after flea re-infestations was 98.0-100% for the 12 weeks of the study. Except for 4 hours after the 12-week flea re-infestation, flea reduction was significant for all time points after flea re-infestation.

**Conclusions:**

Single oral fluralaner administration rapidly eliminates existing flea infestations and provides excellent protection against fleas over 12 weeks following treatment.

## Background

The cat flea, *Ctenocephalides felis*, is the main flea species infesting dogs and cats [1] and the most important ectoparasite for dogs in many parts of the world [2]. A persistent flea infestation without treatment can provoke intense pruritus, self-inflicted trauma and even, in case of massive infestation, anemia [2]. In some dogs, flea exposure leads to the more serious condition of flea allergy dermatitis (FAD), a result of hypersensitivity to flea saliva components [1]. Once sensitization has occurred, relapse of lesions can be initiated by just a small number of flea bites. Clinical signs are usually transient, but chronic skin changes might occur, like alopecia, crusts, hyperpigmentation and lichenification, if the flea infestation is not eliminated [3]. Dogs are affected by the adult fleas only. Other flea life-cycle stages contaminate their environment, where flea development from egg via larvae and pupae to newly emerged adult fleas takes place. Elimination of adult fleas before they start laying eggs is essential for successful flea control [4]. Approximately 24 hours are needed between the flea’s first blood meal and the start of laying eggs [1]. Thus, it is necessary to effectively kill all adult fleas within this time frame.

The active ingredient of Bravecto™, fluralaner, is a member of the isoxazoline class, a novel class of antiparasitic drugs that inhibits γ-aminobutyric acid (GABA)- and glutamate-gated chloride channels with significant selectivity for insect neurons over mammalian neurons [5,6]. Fluralaner formulated as a flavoured chewable tablet is commercially available for immediate and persistent killing of ticks and fleas on dogs over 12 weeks, eliciting its primary action through feeding activity [7]. In a field trial, a single dose of fluralaner provided 12 weeks activity against fleas in dogs [8], and it was shown to be safe and well tolerated [8,9].

The aim of the current studies was to determine a) how fast fluralaner starts to kill fleas (within 0.5-4 hours post-treatment) and b) the speed of flea kill within the 4–24 hours after infestation over the 12-week efficacy duration of fluralaner.

## Methods

### Study set-up

Three blinded, randomized, negative controlled studies were performed, one start to kill study in South Africa and two speed of kill studies in Germany. The start to kill study was conducted in accordance with Good Clinical Practice (VICH guideline GL9, Good Clinical Practice (EMA, 2000)), in compliance with the South African National Standard “SANS 10386:2008: The care and use of animals for scientific purposes” and ethical approval was obtained by the ClinVet Animal Ethics Committee (CAEC) before the study start. The 2 speed of kill studies were conducted in accordance with the OECD Principles of Good Laboratory Practice (GLP) and the GLP Principles of the German “Chemikaliengesetz” (Chemicals Act), in compliance with the German animal welfare regulations and ethical approval was obtained before the start of the study.

For each flea assessment time point in each of the three studies, 6 treated dogs and 6 control dogs were included, thus in total 96 dogs (52 male and 44 female). All dogs included were over 6 months of age, weighed between 8.9 and 18.8 kg, and were mixed or pure bred (Beagles). Each dog was in good health, had not been treated with any parasite control product within 3 months prior to a 7 day acclimatization period, and was uniquely identified by a microchip number. In the start to kill study and the speed of kill studies, laboratory bred flea strains, both originating from Germany and collected from the field less than 10 years prior to the studies, were used.

Within the acclimatization period, the flea susceptibility of each dog was confirmed by a flea infestation with 100 (± 4) fleas (start to kill) or 80 fleas (speed of kill), followed by flea removal and count 24 (± 2) hours later. All dogs included in the study harboured more than 50% of the number of originally infested fleas. Ranking of the dogs was performed within gender (start to kill study) or without gender separation (speed of kill studies) by descending flea counts and dogs were randomly allocated to study groups using a computer generated randomization list. In the start to kill study, dogs were housed individually. In the speed of kill studies, dogs were group-housed within their corresponding study group during periods without flea infestation. On days when dogs carried fleas, all dogs were individually housed.

### Treatment

One or 2 days before treatment, all dogs were infested with either 100 (± 4) (start to kill study) or 80 (speed of kill studies) unfed adult *C. felis* fleas. On the day of treatment (day 0), each dog received half of its daily food ratio approximately 20 minutes before treatment and the balance directly after treatment. Dogs in the treatment groups were treated orally with fluralaner chewable tablets (Bravecto™), based on the dog’s individual body weight, to achieve a minimum dose of 25 mg fluralaner/kg body weight. The chewable tablet was administered by placement in the back of the oral cavity over the tongue to initiate swallowing. Each treated dog was continuously observed for 1 hour after administration to monitor for vomit or tablet/s spit out, which did not occur in any of the 3 studies. Control group dogs remained untreated. General health observations were performed daily throughout the complete study period in all 3 studies.

### Flea infestations and assessments

In the start to kill study, all dogs were infested with 100 (± 4) fleas 1 day before treatment. Flea counts were performed with one pair of treatment plus control group at either 0.5 (± 5 min), 1 (± 15 min), 2 (± 15 min), or 4 (± 15 min) hours after fluralaner treatment. In the two speed of kill studies, dogs were infested with 80 fleas on days −2, 28 (4 weeks), 56 (8 weeks) and 84 (12 weeks). Flea counts were performed with one pair of treatment plus control group at either 4 and 8 (± 0.75) hours in one study or at 12 and 24 (± 1.5) hours in the other study after fluralaner treatment (week 0) or each flea re-infestation (weeks 4, 8, and 12). Each dog was combed to remove and count live adult fleas. Personnel conducting comb counts were blinded with regard to study group.

### Efficacy evaluation

The individual dog was the experimental unit in all statistical calculations. Data from each flea count time point were analysed separately. Significant differences were assessed between the log-counts of adult live fleas in each treated group at each assessment time point in comparison to the log-counts of the respective untreated control group. Study groups were compared using a linear mixed model including study group as a fixed effect and block as a random effect. The two-sided level of significance was declared when *P* ≤ 0.05 (SAS Institute Inc., Cary, NC, USA, release 9.2).

Efficacy was calculated using geometric means with Abbott’s formula:

Efficacy (%) = 100 x (M_C_ - M_T_)/M_C,_ where M_C_ was the mean number of total adult live fleas on untreated dogs and M_T_ the mean number of total adult live fleas on treated dogs. In case of zero counts, the geometric mean (of fleas) was calculated as follows:

$$ {\mathrm{X}}_{\mathrm{g}}={\left({\displaystyle \prod_{\mathrm{i}=1}^{\mathrm{n}}\left({\mathrm{x}}_{\mathrm{i}}+1\right)}\right)}^{\frac{1}{\mathrm{n}}}-1 $$

## Results

No adverse event considered to be related to oral fluralaner treatment was observed in any dog.

In the start to kill study (Table 1), the fluralaner activity against fleas had started already at 1 hour post treatment (8% numerical efficacy). Significant flea reduction was observed at 2 and 4 hours (36.7% and 88% efficacy). One dog without any fleas and three dogs with ≤ 6 fleas were found 4 hours after treatment.

**Table 1:** **Start to kill study: mean flea counts, flea count range and percentage efficacy 0.5, 1, 2, and 4 hours after a single oral administration of fluralaner to dogs**

**Time post treatment**	**0.5 hour**		**1 hour**		**2 hours**		**4 hours**	
**Study Group**	**Fluralaner**	**Control**	**Fluralaner**	**Control**	**Fluralaner**	**Control**	**Fluralaner**	**Control**
**Mean** ^**a**^ **flea counts (n)**	78.71	73.96	78.14	84.89	48.70	76.97	7.38	61.57
**Count range (n)**	74-86	65-89	47-96	64-97	33-69	61-99	0-80	30-80
**Efficacy (%)**	0.0%		8.0%		36.7% ^b^		88.0% ^b^	

^a^Geometric mean.

^b^Log-counts of live fleas from the treated group were significantly different (p ≤ 0.05) from log-counts of the respective untreated control group.

In the speed of kill studies (Table 2), the efficacy against fleas after fluralaner treatment was 80.5% at 4 hours and remained ≥ 99.4% at 8, 12 and 24 hours.

**Table 2:** **Speed of kill studies: mean flea counts, flea count range and percentage efficacy over 12 weeks after a single oral administration of fluralaner to dogs**

**Assessment time points** ^**a**^		**4 hours**		**8 hours**		**12 hours**		**24 hours**	
**Study Group**		**Fluralaner**	**Control**	**Fluralaner**	**Control**	**Fluralaner**	**Control**	**Fluralaner**	**Control**
**Week 0**	**Mean** ^**b**^ **flea counts (n)**	4.14	21.21	0.30	49.93	0.0	73.59	0.0	13.85
	**Count range (n)**	0-21	1-71	0-4	6-80	0	58-79	0	0-75
	**Efficacy (%)**	80.5%^c^		99.4%^c^		100%^c^		100%^c^	
**Week 4**	**Mean** ^**b**^ **flea counts (n)**	2.38	74.78	0.0	77.79	0.0	73.53	0.0	69.35
	**Count range (n)**	0-13	69-80	0	73-80	0	66-80	0	61-75
	**Efficacy (%)**	96.8%^c^		100%^c^		100%^c^		100%^c^	
**Week 8**	**Mean** ^**b**^ **flea counts (n)**	5.71	66.61	0.0	75.01	0.12	69.56	0.0	71.48
	**Count range (n)**	0-43	60-77	0	62-80	0-1	59-80	0	65-79
	**Efficacy (%)**	91.4%^c^		100%^c^		99.8%^c^		100%^c^	
**Week 12**	**Mean** ^**b**^ **flea counts (n)**	45.91	69.02	1.49	74.24	1.02	76.44	0.0	73.25
	**Count range (n)**	15-75	64-75	0-14	67-77	0-16	72-80	0	68-78
	**Efficacy (%)**	33.5%		98.0%^c^		98.7%^c^		100%^c^	

^a^Assessment for fleas xy hours after treatment or re-infestation following treatment.

^b^Geometric mean.

^c^Log-counts of live fleas from the treated group were significantly different (p ≤ 0.05) from log-counts of the respective untreated control group.

After flea re-infestations in weeks 4, 8 and 12 the efficacy at 4 hours was 96.8, 91.4, and 33.5%, respectively. Efficacy at 8, 12 and 24 hours after flea re-infestations was 98.0-100% for the 12 weeks of the study. Only a few or no fleas were observed on the dogs at these time points. Except for 4 hours after the 12-week flea re-infestation, flea reduction was significant for all time points after treatment and re-infestation.

## Discussion

Fluralaner (Bravecto™) is the first orally administered ectoparasiticide to demonstrate an efficacy against fleas over 12 weeks [7]. In the start to kill study, the results show that orally administered fluralaner starts to kill present fleas on the dog already 1 hour after treatment. At 4 hours after treatment 88%, and at 8 hours after treatment 99.4% of the fleas were killed. This pronounced efficacy at 8 hours post treatment was maintained for the full 12-week post treatment period. This result is consistent with the close to 100% efficacy observed at multiple time points in a European field trial [8]. In that field trial, in a fipronil-treated group the efficacy was lower at all time points and required repeated monthly treatment to reach an efficacy comparable to the one of fluralaner after one administration. The pronounced flea efficacy of fluralaner at 8 hours for a period of at least 12-weeks post treatment contributes to the control of the flea population in the environment by the gradual elimination of flea stages. Female fleas start to lay eggs within 24–48 hours after the initiation of the blood meal, which is necessary for egg production and maturation in fleas [10]. By killing all newly emerged adult fleas before they start laying eggs over the extended period of time of 12 weeks, the flea life cycle will be disrupted, i.e. larvae, pupae and new adults will be eradicated from the dog’s environment. The long lasting flea efficacy is strengthened by providing an additional effect on flea reproduction, because fluralaner directly reduces the pupal development and hence emergence of new adult fleas [11]. Therefore, treatment with fluralaner not only has an effect on the existing flea population on the dog, it also contributes to the disruption of the flea life cycle and thus to the extinction of flea burden in an infested environment. Due to the quick onset of activity of fluralaner, fewer fleas are able to bite, which supports the treatment of FAD [8], even to an extent that no further concomitant medication is required [unpublished observations]. A common factor for insufficient ectoparasite control in dogs is poor owner compliance with required monthly re-treatment protocols for existing ectoparasite- therapeutic options [12,13]. Poor owner compliance may also encourage resistance in the flea population by selecting for insecticide-tolerant individuals [13]. A long re-treatment interval and greater owner compliance could reduce the potential for resistance development. Therefore, an active ingredient with a long re-treatment interval, such as fluralaner, leads to better flea control.

## Conclusions

A single oral administration of fluralaner to dogs, formulated as chewable tablet (Bravecto™), leads to an onset of flea killing activity starting at 1 hour and a flea efficacy 98-100% at 8 hours after treatment or following re-infestation throughout a 12-week post-treatment period. Fluralaner effectively kills fleas within a few hours after host infestation, thus contributing to the disruption of the flea life cycle, leading to a depletion of the environmental flea population. This also supports the treatment of FAD. The long re-treatment interval of fluralaner (Bravecto™) offers more convenience over monthly flea-control treatments with a potential compliance advantage.
